# Effects of foliar application of micronutrients on concentration and bioavailability of zinc and iron in wheat landraces and cultivars

**DOI:** 10.1038/s41598-021-02088-3

**Published:** 2021-11-23

**Authors:** Baozhen Hao, Jingli Ma, Lina Jiang, Xiaojie Wang, Yongqu Bai, Chuangchuang Zhou, Simin Ren, Chunxi Li, Zhimin Wang

**Affiliations:** 1 School of Life Science and Basic Medicine, Xinxiang University, Xinxiang, 453003 Henan China; 2 College of Life Sciences, Henan Normal University, Xinxiang, 453007 Henan China; 3 College of Agronomy, China Agricultural University, Beijing, 100193 China

**Keywords:** Physiology, Plant sciences

## Abstract

Foliar application of micronutrient is a rapid and promising strategy to enhance the concentration and bioavailability of micronutrients in wheat grain. To explore the effects of foliar application of micronutrients on the concentration and bioavailability of zinc and iron in grain in wheat cultivars and landraces, field experiments were carried out using 65 wheat cultivars and 28 landraces to assess the effects of foliar application of zinc (iron) on phytic acid concentrations, zinc (iron) concentrations and their molar ratios. The results indicated that mean grain zinc concentration of landraces (44.83 mg kg^−1^) was 11.13% greater than that of cultivars (40.34 mg kg^−1^) on average across seasons, while grain iron concentration did not differ significantly between landraces (41.00 mg kg^−1^) and cultivars (39.43 mg kg^−1^). Foliar zinc application significantly improved the concentration and bioavailability of zinc in grains in both cultivars and landraces, while landraces had almost two-fold more increase in grain zinc and also greater improvement in zinc bioavailability compared to cultivars. While foliar iron application did not significantly affect iron concentration and bioavailability in grains in either cultivars or landraces. Our study showed that, with foliar application of zinc but not iron, wheat landraces had better performance than cultivars in terms of the increases in both concentration and bioavailability of micronutrient in grains.

## Introduction

Micronutrient deficiency in humans is widespread and is estimated to affect over three billion people worldwide^1^. Zinc and iron deficiencies are the most prevalent micronutrient deficiencies, and approximately 17% and 33% of the world’s population face zinc and iron deficiency, respectively, predominantly pre-school children and pregnant women in low- and middle-income countries^2,3^. It has been estimated that about 500,000 children under five years of age die each year due to zinc and iron deficiencies^4^. Zinc and iron deficiencies are also serious health problems in China^5^, and it is estimated that around 340 million people suffer from iron deficiency anemia^6^ and over 100 million people are at risk of zinc deficiency^7^. High consumption of cereal based foods with low concentration and poor bioavailability of zinc and iron is considered to the main driver of zinc and iron deficiency in humans^8^. Wheat (*Triticum aestivum* L.) as an important cereal crop is the most important source of calories and protein, as well as essential micronutrients such as zinc and iron for the majority of people in developing countries^9^. China is the largest wheat producer and consumer in the world, and for the majority of people in China, wheat-based foods are the major dietary component and supply 20.1% of dietary zinc and 25.6% of iron^5^. So biofortifying wheat with zinc and iron is an effective approach to diminish zinc and iron malnutrition in China and also in other developing countries.

Conventional plant breeding is a major tool that is used for biofortification of cereals with essential mineral elements^8^. Large germplasm resources are the basis of conventional breeding^10^. The history of wheat cultivation in China lasted about four millennia^11^, and there are large wheat germplasm resources such as landraces, which is a valuable resource for wheat breeding^12^. Previous studies have indicated the existence of substantial variation for grain zinc and iron concentrations in wheat landraces^13–16^, and relative to modern wheat cultivars, landraces contained higher concentrations of zinc and iron in grains^8,17,18^. Therefore, exploiting the rich genetic diversity for zinc and iron concentrations in wheat landraces offers promising opportunities for genetic biofortification of zinc and iron in wheat^17,19^.

Application of micronutrient fertilizers is a rapid and promising strategy for biofortification of wheat with micronutrient^9,20,21^. Generally, micronutrient fertilizers can be applied to soils or sprayed onto foliage^22^. It is well known that both foliar and soil application of zinc is effective in improving the concentration and bioavailability of zinc in wheat grains^23^, and recent studies showed that, in terms of achieving high concentrations and bioavailability of zinc in grains, foliar zinc applications were much more effective than soil applied zinc^24–26^. In the case of iron, it seems that foliar application is only a little more effective than soil application in improving the iron concentration in wheat grain^23^. Several studies compared the effect of zinc and iron fertilizers in terms of enrichment of wheat grains, and found soil and/or foliar iron fertilizers were less effective than application of zinc^9,23,27^.

The effect of foliar application of micronutrients on the concentration and bioavailability of zinc and iron in grains in wheat cultivars has been widely studied, little is known about its effect on wheat landraces. The objectives of this study were (1) to compare the grain concentration and bioavailability of zinc and iron in wheat landraces as affected by foliar application of micronutrients, and (2) to evaluate the response of the wheat landraces to foliar application of micronutrients compared to modern wheat cultivars.

## Materials and methods

### Plant material

A total of 93 wheat (*Triticum aestivum*) accessions were used in this study, including 28 landraces and 65 cultivars (Table S1). These 93 accessions were from a mini core collection of Chinese wheat accessions which is estimated to represent approximately 70% of genomic diversity of Chinese wheat^11^. The experimental research on plants conducted in this study complies with relevant institutional, national, and international guidelines. The field studies were carried out in accordance with local legislation.

### Experimental site

**Figure 1:**
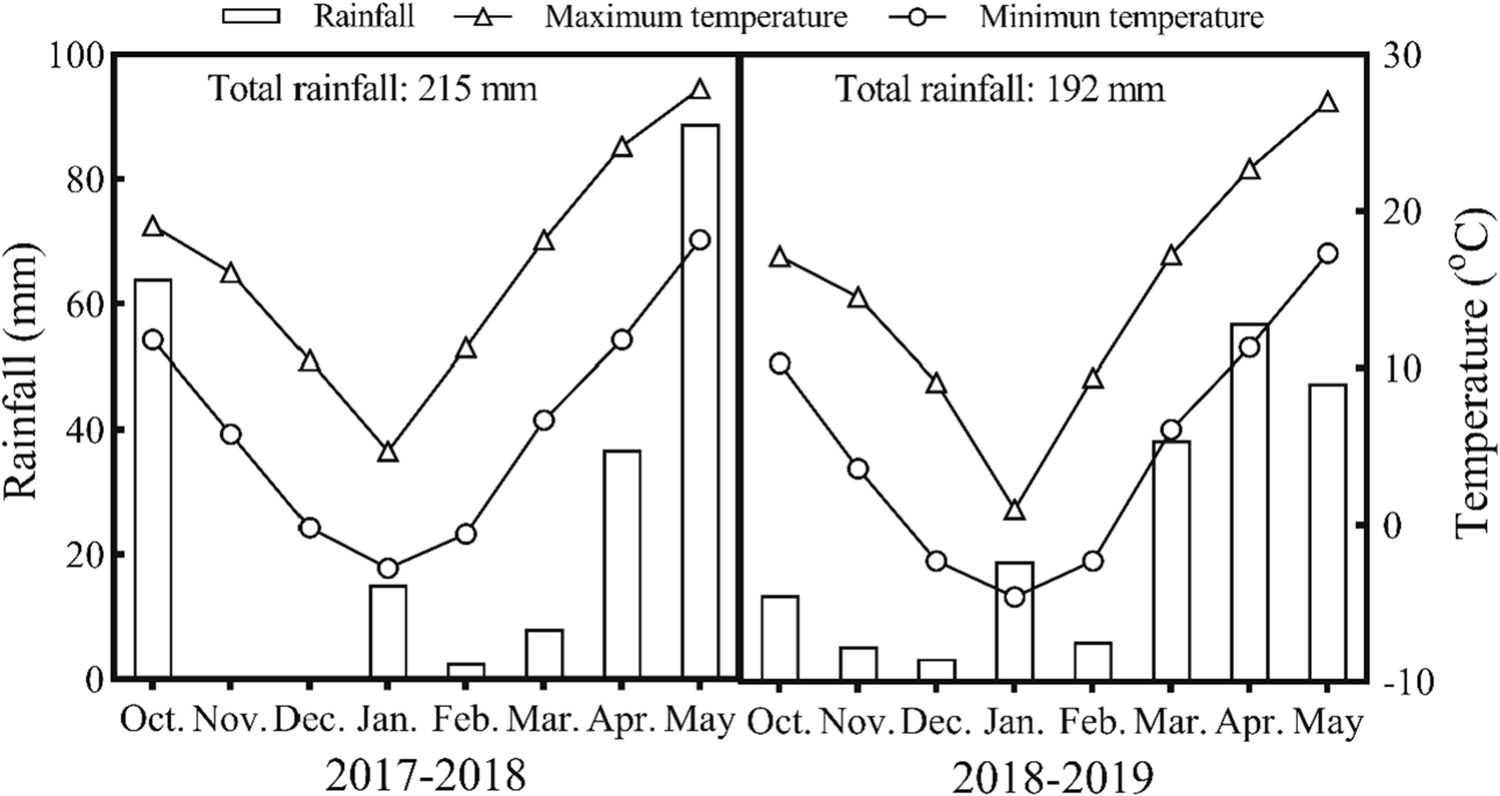
Monthly and annual rainfall and average maximum temperature and minimum temperature during the 2017/18 and 2018/19 wheat growing seasons at Xinxiang, China.

Field experiments were carried out at experimental station (35.2°N, 113.8°E) of Henan Normal University (Xinxiang, Henan province, China). The chemical properties of the 0 to 30 cm soil layer were as follows: pH 8.0, 12.2 g kg^−1^ of organic matter (Walkley–Black method), 65.1 mg kg^−1^ of alkaline hydrolysis N (Kjeldahl method), 112.7 mg kg^−1^ of available K (Dirks-Sheffer method), 9.1 mg kg^−1^ of Olsen P (Olsen method), 0.98 mg kg^−1^ of DTPA-extractable zinc, and 5.48 mg kg^−1^ DTPA-extractable iron. DTPA extractable zinc and iron were determined with the method described by Lindsay and Norvell (1978)^28^. Meteorological data during wheat growing season was collected from an automatic weather station located at the experimental site (Fig. 1).

### Experimental design and treatments

Experiment I was performed during the 2017/18 and 2018/19 wheat growing seasons and was arranged following a randomized complete block design with three replications. Each experimental plot was 1.0 m wide and 2.0 m long with a row spacing of 0.20 m. In each season, 93 wheat accessions were sown using a hand-operated seeder and seeding rates were adjusted for each accession to achieve a target population of 300 plants m^−2^. Prior to sowing, urea at 217 kg ha^−1^, triple superphosphate at 270 kg ha^−1^ and potassium sulfate at 210 kg ha^−1^ were surface broadcast-applied by hand and incorporated into the top soil (0–0.30 m) by rotary tiller, and topdressing N (urea at 109 kg ha^−1^) was broadcast-applied at stem elongation stage followed by irrigation. Surface irrigation was carried out using movable pipelines before sowing, at jointing and anthesis. Herbicide and insecticides were applied when necessary to control insect damages and weeds.

Experiment II was performed during the 2018/19 wheat growing season and was set up in a randomized complete block design with three replications. Ninety-three wheat accessions (Table S1) were analysed in Experiment II. Three treatments were used, untreated control (-Zn, foliar application of distilled water) and foliar application with zinc (+Zn). Each experimental plot comprised five rows, 1.0 m wide and 4.0 m long, with half the plots (1.0 m × 2.0 m) were fertilized with zinc and the other half plots received no zinc fertilizer. Zinc fertilizers were supplied with 0.5% (w/v) of aqueous solution of ZnSO_4_·7H_2_O, and for control plots, distilled water was applied. During the season, foliar sprays were applied at anthesis, 12 d and 21d after anthesis, and each spray was performed in the very late afternoon to avoid possible leaf damage due to more salts accumulation on sunny day and at high day temperatures. At each time of foliar application, a solution volume of 1000 L ha^−1^ was used for each treatment. Wheat seeding and field management were the same as experiment I.

Experiment III was carried out in 2018/19 season using 28 landraces and 65 cultivars (Table S1). The design of the experiment was a randomized complete block design with three replications. Each experimental plot was 1.0 m wide and 4.0 m long with a row spacing of 0.20 m. The treatments included nil iron (–Fe, foliar application of distilled water) and foliar iron application (+ Fe), with half the plots (1.0 m × 2.0 m) were fertilized with iron and the other half plots were supplied with distilled water. Iron fertilizers were supplied with 0.3% (w/v) of aqueous solution of FeSO_4_·7H_2_O. Foliar sprays were applied at anthesis, 12 d and 21d after anthesis, and each spray was performed in the very late afternoon. At each time of foliar application, a solution volume of 1000 L ha^−1^ was used for each treatment. Wheat seeding and field management were the same as experiment I.

### Grain sampling

At physiological maturity in all three field experiments, plants were harvested manually from three center rows of each plot to determine grain yield, which was corrected to 13% moisture content. Subsamples of about 100 g of grain were washed with tap water and deionized water, respectively, and then oven dried at 65℃until constant weight was reached. These oven-dried subsamples were then ground with a stainless steel grinder (MM400, Retsch, Haan, Germany) and stored in sealed plastic bags for further chemical analysis.

### Zinc, iron and phytic acid determination

In Experiment I, II and III, grain zinc and iron concentrations were measured for three replicates of each treatment. For the analysis of grain zinc and iron, dried and ground sample was digested with a mixture of 4.0 ml HNO_3_ and 1.0 ml H_2_O_2_ using a microwave accelerated reaction system (CEM, Mars 6, Matthews, NC, USA). The zinc and iron concentrations in the digested solutions were determined by Inductively Coupled Plasma Optical Emission Spectrometry (ICP-OES, iCAP 7000, Thermo Fisher Scientific, Germany). A certified standard reference material (No. GBW10011), provided by Institute of Geophysical and Geochemical Exploration, was used as the quality control sample. For both zinc and iron, the averaged recovery rates were more than 95% and relative standard deviation were less than 5%. The grain concentrations of zinc and iron were expressed on the basis of oven-dried weight.

In Experiment II and III, the phytic acid concentration of wheat grain was determined for three replicates of each treatment, according to the method described by Chen et al. (2008)^29^. 30 mg of dried samples were weighed in a 2 ml screw top centrifuge tube and extracted with 1 ml of 0.4 M HCl–15% TCA at room temperature for 3 h with continuous stirring. The samples were then centrifuged at 3000×g for 15 min, 25 μl of the supernatant were mixed with 275 μl of 36.3 mM NaOH and 100 μl of 0.03% (w/v) FeCl_3_·6H_2_O–0.3% sulfosalicylic acid. The mixture was centrifuged for 10 min at 2000×g, 200 μl supernatant was transferred into a new 96-Well plate. The absorbance was measured using a multimode plate reader (EnSpire, Perkin Elmer, USA).

### Phytic acid:zinc (PA:Zn)and phytic acid:iron (PA:Fe)molar ratios

The PA:Zn and PA:Fe molar ratios were considered as the bioavailability of zinc and iron in grains, respectively^30^. The ratios were calculated as follow^30^:

1 $$ {\text{PA}}{:}{\text{Zn molar ratio}} = \frac{{65.40 \times {\text{Phytic acid concentration in mg}}\, {\text{g}}^{ - 1} }}{{\left( {660.04 \times {\text{Zinc concentration in mg}}\, {\text{kg}}^{ - 1} /1000} \right)}} $$

2 $$ {\text{PA}}{:}{\text{Fe molar ratio}} = \frac{{55.85 \times {\text{Phytic acid concentration in mg}} \,{\text{g}}^{ - 1} }}{{\left( {660.04 \times {\text{Iron concentration in mg}} \,{\text{kg}}^{ - 1} /1000} \right)}} $$

where 660.04, 65.40, and 55.85 g mol^−1^ were molecular weight of phytic acid, zinc and iron, respectively.

### Statistical analysis

R software v 3.5.3 (R Core Team, 2019), SAS v.9.2 (SAS Institute Inc., Cary, NC, USA), and Sigma Plot 13.0 (Systat Software, Inc., San Jose, CA, USA) were used for statistical analyses. Descriptive statistical analysis was performed using Sigma Plot 13.0 software. The histograms were developed for grain zinc and iron concentrations in both experiments. Pearson correlation coefficients were used to analyse the relationships among the measured traits. Analysis of variance was conducted through General Linear Model procedure by using the correct error to evaluate each factor and interaction. The variety, zinc treatment, iron treatment, and their interactions were treated as fixed effects. Replication was considered random effect. Mean values were compared by the least significant differences test. Broad sense heritability (H^2^) was estimated for grain zinc and iron concentrations across two growing seasons using variance components calculated based on ANOVA, using the formula H^2^ = σ^2^_G_ / (σ^2^_G_ + σ^2^_GE_/y + σ^2^_R_/ry), where σ^2^_G_ is the genotypic variance, σ^2^_GE_ is the genotype × environment variance, and σ^2^_R_ is the residual error variance for r replicates and y years.

## Results

### Concentrations and yields of grain zinc and iron

**Figure 2:**
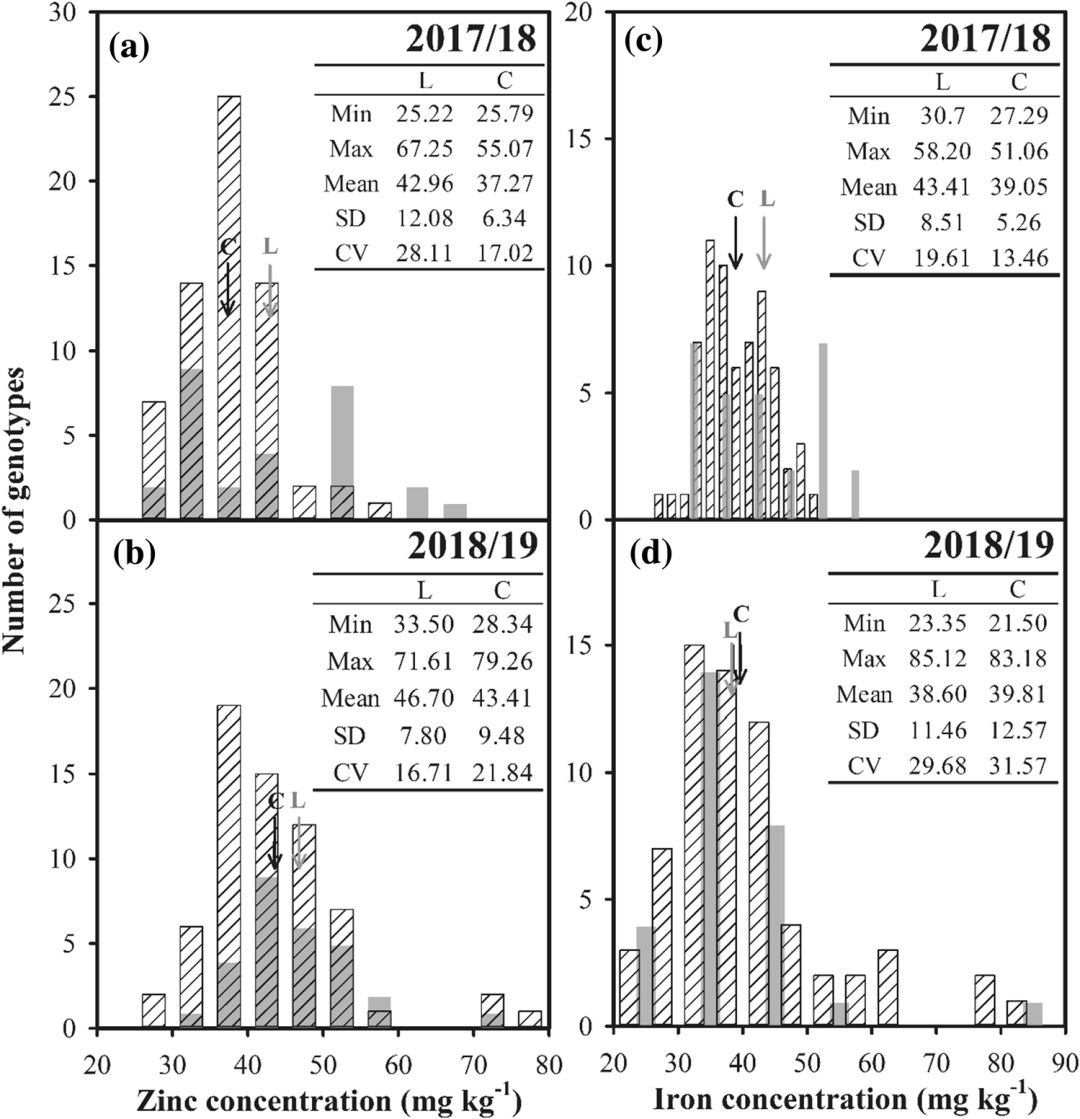
Frequency distribution of grain (**a,b**) zinc and (**c,d**) iron concentrations of the ninety-three wheat accessions in the Experiment I. The landraces are marked in grey. Arrows indicate mean values of (**L**) landrace and (**C**) cultivar.

**Figure 3:**
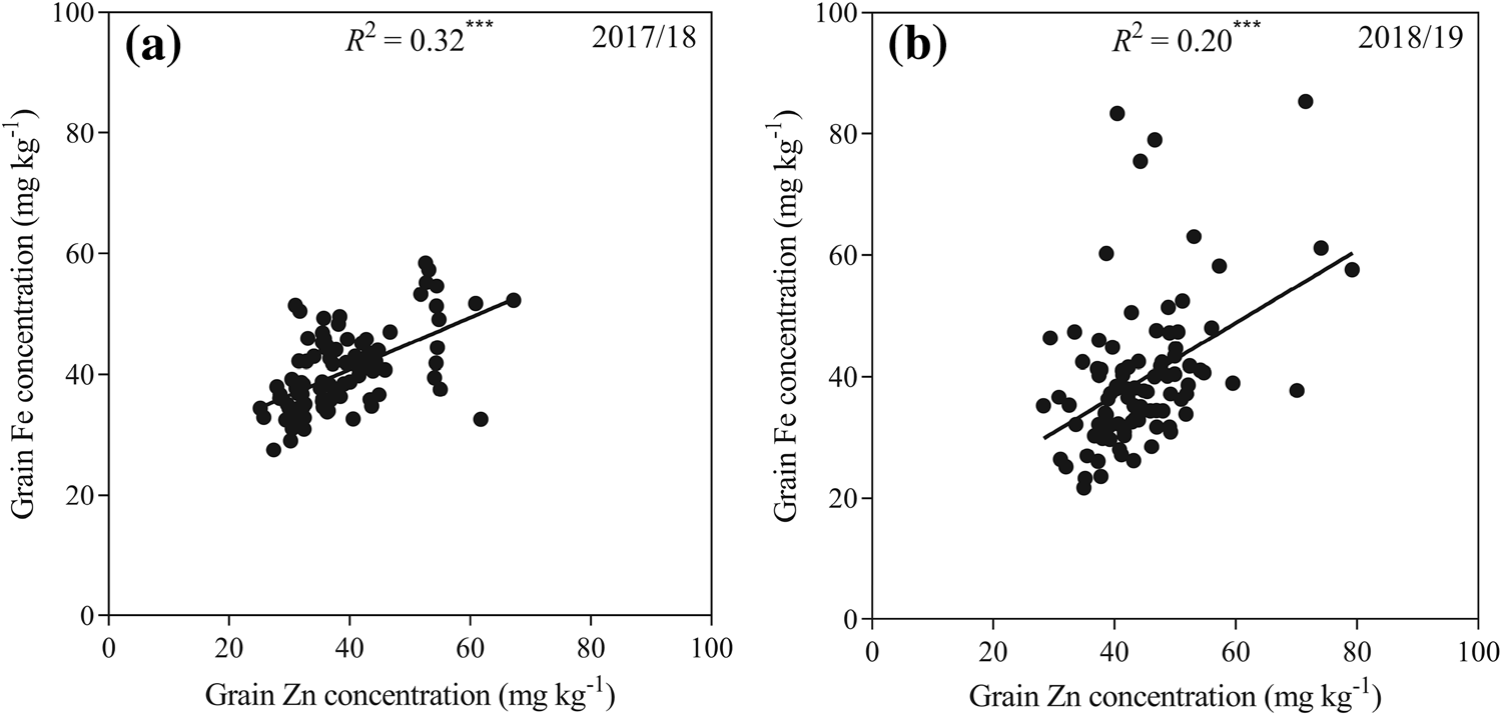
Correlations between Zn and Fe concentration in grains of 93 wheat accessions in (**a**) 2017/18 and (**b**) 2018/19 wheat growing seasons. Note: ^∗∗∗^ indicates statistically significant at *P* < 0.001 level.

The variations of grain zinc and iron concentrations were studied across two seasons in 28 Chinese wheat landraces and 65 cultivars. Frequency distributions of grain zinc and iron concentration, with landrace indicated in grey, are presented in Fig. 2. In both seasons, the cultivar accessions showed normal distributions for both zinc and iron concentrations, with the exception of iron, which did not distribute normally in the 2018/19 season. Also, significant and positive correlations were found between zinc and iron concentration in both seasons (Fig. 3).

In the 2017/18 season, both grain zinc and iron concentrations were greater (*P* < 0.05) for landraces than for cultivars (Fig. 2a,c). However, there were no significant differences in both grain zinc and iron concentrations between landraces and cultivars in the 2018/19 season (Fig. 2b and d). Averaged across seasons, grain zinc concentration for landraces was 44.83 mg kg^−1^, which was 11.13% higher (*P* < 0.05) than that for cultivars (40.34 mg kg^−1^), while grain iron concentration did not differ significantly between landraces (41.00 mg kg^−1^) and cultivars (39.43 mg kg^−1^). Furthermore, both zinc and iron distributed across wider ranges in the 2018/19 season than in the 2017/18 season. Also, as compared with cultivars, the range of zinc distribution for landraces was wider in the 2017/18 season but narrower in the 2018/19 season. While the ranges of iron distribution were similar for cultivars and landraces in each seasons.

**Figure 4:**
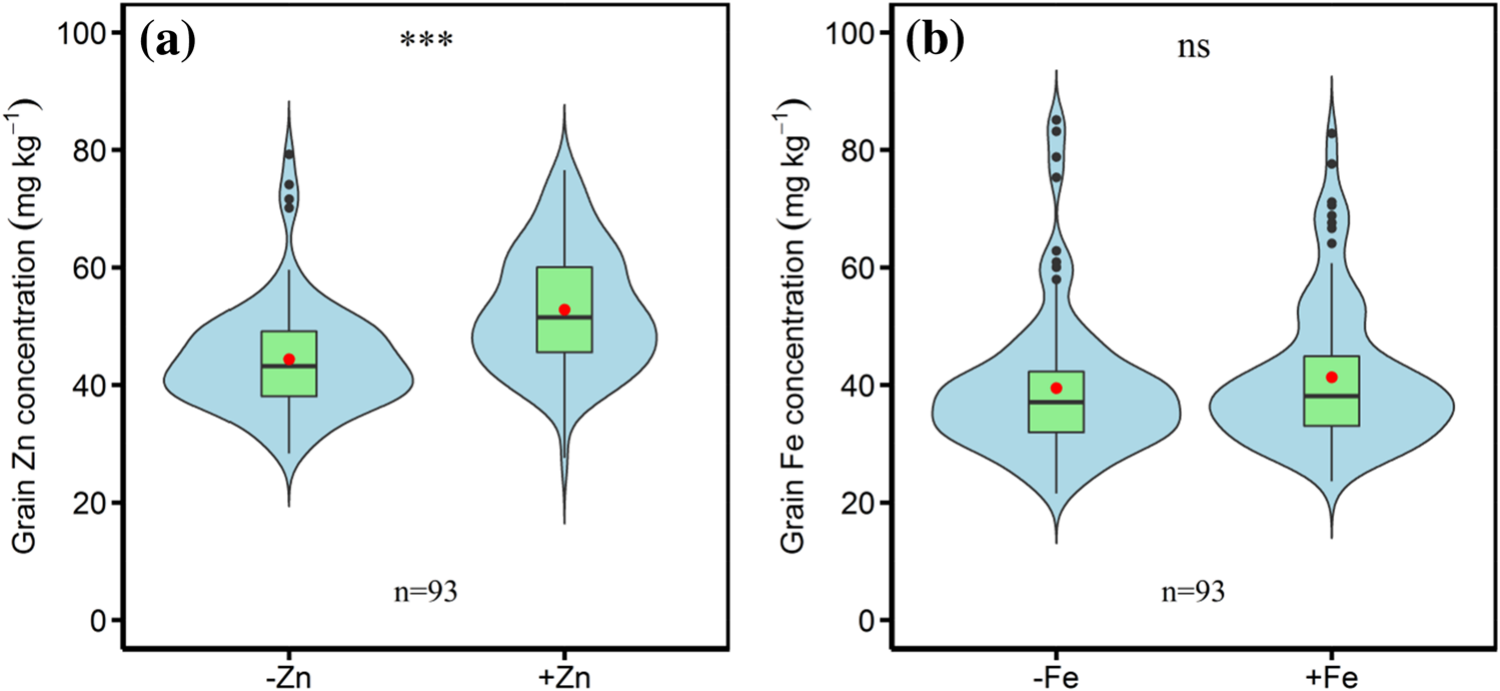
Effects of foliar application of micronutrients on the grain concentrations of (**a**) zinc and (**b**) iron of the ninety-three wheat accessions. Note: −Zn and + Zn indicate without and with foliar zinc application, respectively, − Fe and + Fe indicate without and with foliar iron application, respectively. The boxes boundaries indicate the 75% and 25% quartiles; whiskers indicate the 95% confidence limits; the black circles indicate outliers; the solid horizontal line and red circle indicate median and mean, respectively. ∗ ∗∗ is statistically significant at *P* < 0.001, ns indicate not significant at *P* < 0.05.

Averaged across genotypes, foliar zinc application significantly increased grain zinc concentration from 44.4 mg kg^−1^ to 52.8 mg kg^−1^ with an increase of 18.9% compared to no zinc application (Fig. 4a). Foliar iron application increased grain iron concentration from 39.4 mg kg^−1^ to 41.3 mg kg^−1^ with an increase of 4.8%, but this increase was not statistically significant (Fig. 4b). The results indicated that, with foliar application of micronutrients, the increase in concentration of grain zinc was much more efficient than that of grain iron.

**Figure 5:**
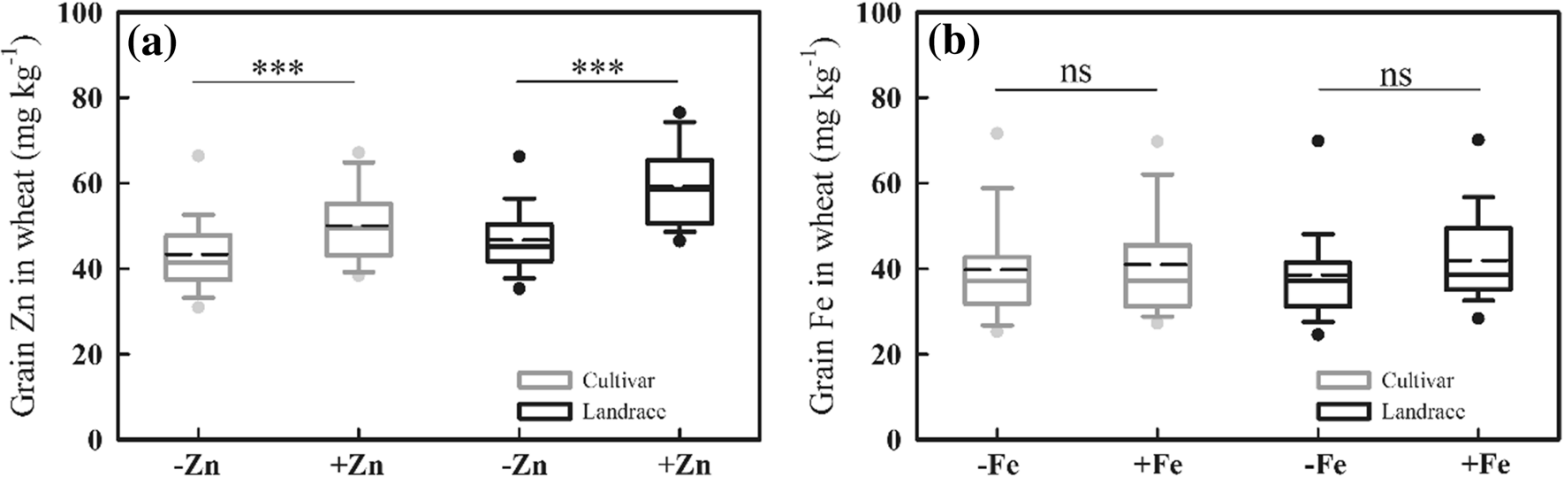
Effects of foliar application of micronutrients on the grain concentrations of (**a**) zinc and (**b**) iron of Chinese wheat landraces (n = 28) and cultivars (n = 65). Note: The box boundaries indicate upper and lower quartiles; the whiskers indicate the 90th and 10th percentiles; the circles indicate the 95th and 5th percentiles; the solid and dashed horizontal lines indicate median and mean, respectively. ∗  ∗  ∗ is statistically significant at *P* < 0.001, ns indicate not significant at *P* < 0.05.

**Figure 6:**
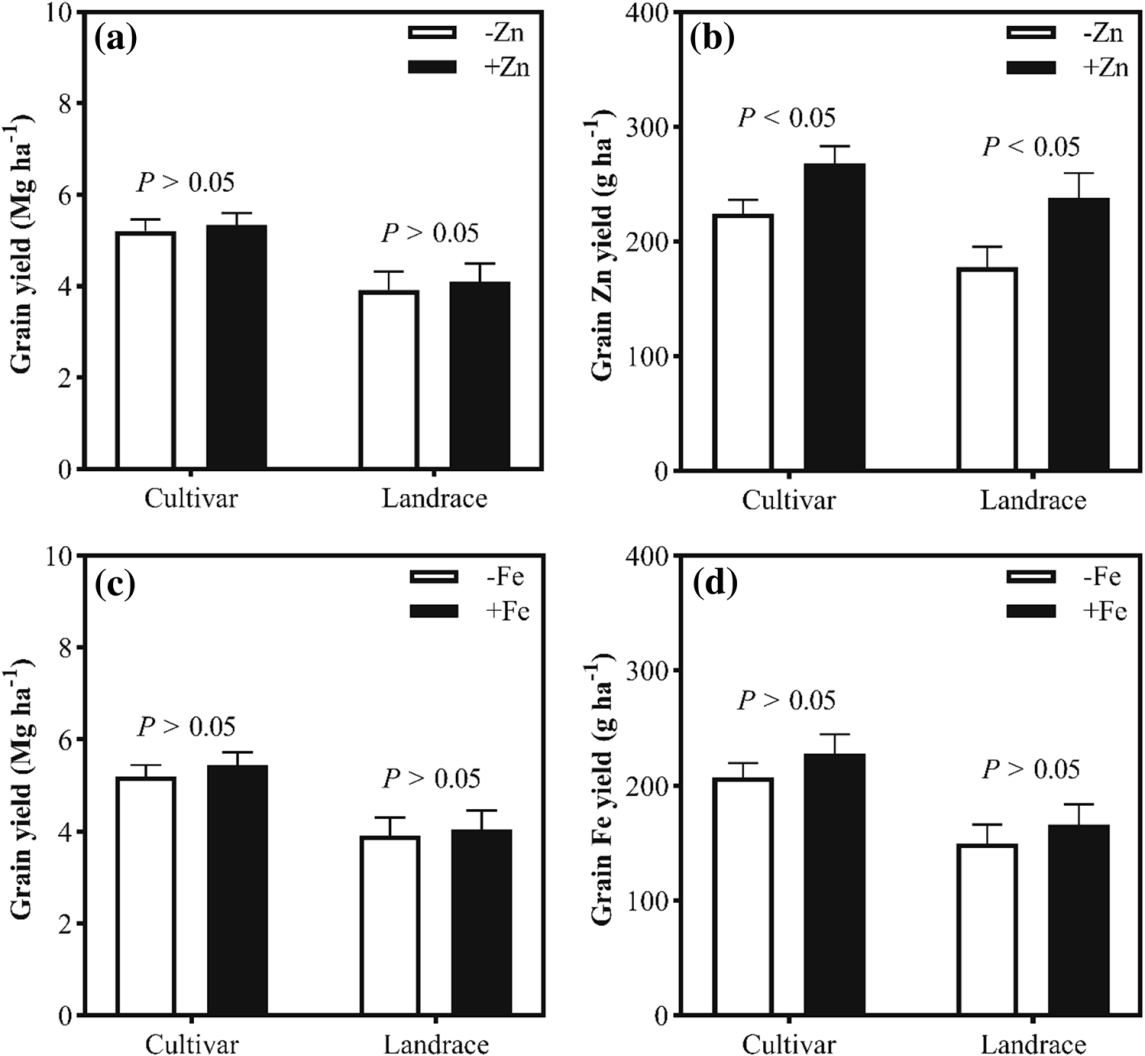
Comparison of (**a**) grain yield and (**b**) grain Zn yield between + Zn and –Zn treatment in Experiment II and (**c**) grain yield and (**d**) grain Fe yield between + Fe and –Fe treatment in Experiment III for wheat cultivars and landraces.

The grain zinc concentration of cultivars and landraces were both significantly increased by foliar application of zinc, and the increase in grain zinc concentration in landrace was 12.5 mg kg^−1^, which was about two-fold greater than that in cultivars (6.6 mg kg^−1^) (Fig. 5a). Also, foliar application of zinc significantly increased grain zinc yield from 224.6 g ha^−1^ to 268.6 g ha^−1^ for cultivars and from 177.9 g ha^−1^ to 238.2 g ha^−1^ for landraces (Fig. 6b) even though it did not significantly increase grain yield of cultivars and landraces (Fig. 6a). The results indicate that, with foliar zinc application, landraces accumulated more zinc in grains than cultivars.

In contrast to foliar zinc application, foliar iron application did increase grain iron concentration from 39.8 to 41.0 mg kg^−1^ for cultivars (3.1%) and from 38.6 to 42.0 mg kg^−1^ for landraces (8.9%), but these increases were not statistically significant (Fig. 5b). Similarly, both grain yield and iron yield of cultivars and landraces were not significantly affected by iron application (Fig. 6c, d). Also, there was no significant interaction between genotype with iron treatment for grain iron concentration (*P* > 0.05) (data not shown). The results suggest that, with foliar iron application, the increase in the concentration and yield of grain iron did not differ between cultivars and landraces.

Pearson correlation analysis was performed in order to analyze the relationships between changes of grain micronutrient concentration and the other studied traits (Table 1). For both wheat cultivars and landraces, changes of grain zinc and iron concentration were poorly correlated (*P* > 0.05) with grain yield. These findings indicate that foliar fertilization of zinc increased zinc in wheat grains despite the yield potential of cultivars and landraces. Also, change of grain iron concentration in both cultivars and landraces showed significant negative correlation (*P* < 0.05) with grain iron concentration under no-iron application. Similar results were also observed for change of grain zinc concentration.

**Table 1:** Pearson correlation coefficients between changes of grain micronutrient concentration and the other studied traits in wheat landraces (n = 28) and cultivars (n = 65) under Zn and no-Zn application in Experiment II and Fe and no-Fe application in Experiment III.

Experiment II	Trait	GY with Zn fertilizer	GY without Zn fertilizer	GZnC with Zn fertilizer	GZnC without Zn fertilizer
Cultivar	GZnC change	0.135	0.088	0.506^b^	− 0.526^b^
Landrace	GZnC change	0.089	0.107	0.611^b^	− 0.341
Experiment III	Trait	GY with Fe fertilizer	GY without Fe fertilizer	GFeC with Fe fertilizer	GFeC without Fe fertilizer
Cultivar	GFeC change	0.046	0.033	0.364 ^b^	− 0.287^a^
Landrace	GFeC change	− 0.108	− 0.185	0.310	− 0.455^a^

Grain Zn concentration (GZnC), grain Fe concentration (GFeC), grain yield (GY).

^a^and ^b^Significant at the 5% and 1% probability levels, respectively.

### Grain phytic acid concentration

Averaged across genotypes, either foliar zinc spray or iron spray did not have a significantly impact on phytic acid concentration in grains (Table 2). Averaged across fertilizer treatments, phytic acid concentration was about 11% greater in landraces than cultivars in each experiment. The results indicate that foliar application of zinc or iron had no effect on reducing phytic acid concentration in wheat grains.

**Table 2:** Effects of foliar application of zinc (Experiment II) and iron (Experiment III) on the grain phytate concentration of Chinese wheat landraces and cultivars.

Experiment II	Phytate concentration (mg g^−1^)			Experiment III	Phytate concentration (mg g^−1^)		
	–Zn	+ Zn	Mean		–Fe	+ Fe	Mean
Cultivar	9.21a	9.10a	9.16B	Cultivar	9.26a	9.05a	9.15B
Landrace	10.45a	10.12a	10.29A	Landrace	10.52a	10.10a	10.31A
Mean	9.58a	9.41a		Mean	9.64a	9.37a	

Within the same row, values with different lowercase letter are significantly different at *P* = 0.05.

Within the same column, means with different uppercase letter are significantly different at *P* = 0.05.

### Phytic acid:zinc (PA:Zn) and phytic acid:iron(PA:Fe) molar ratios

**Figure 7:**
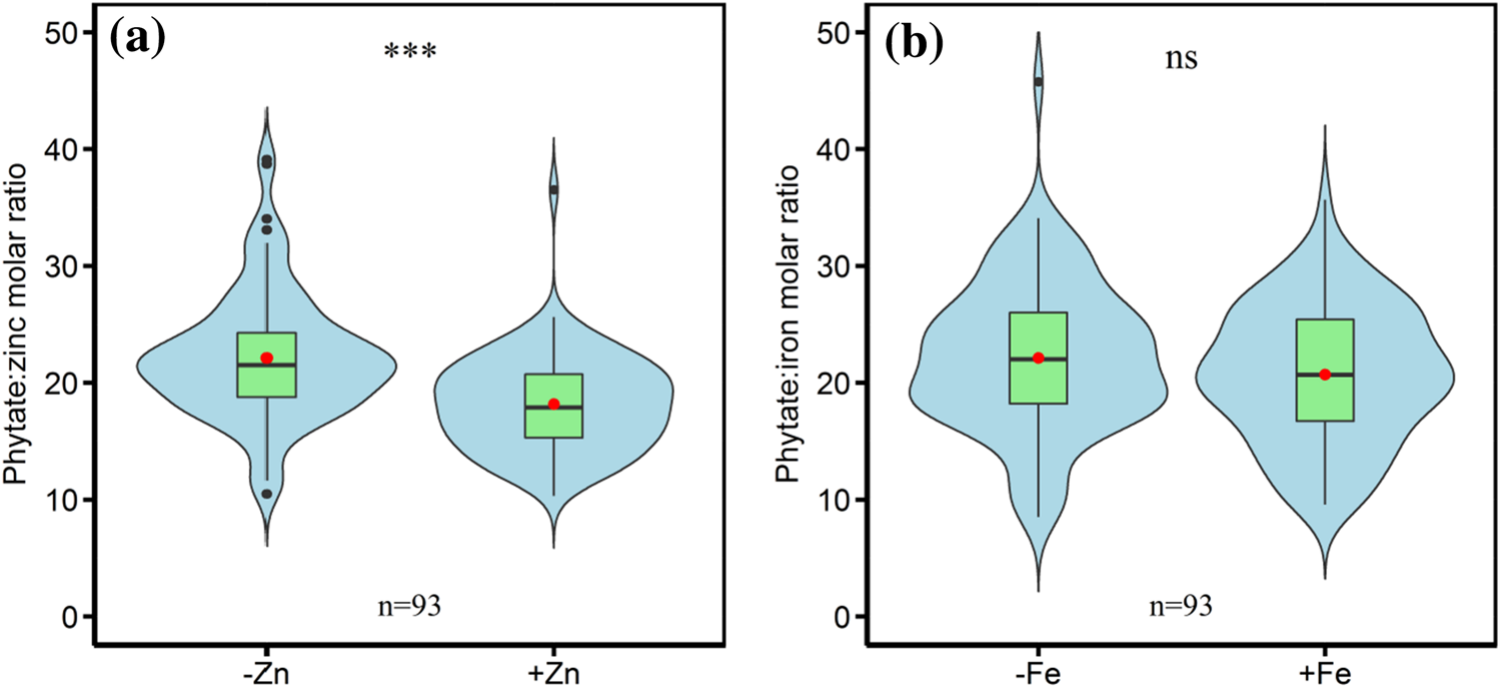
Effects of foliar application of micronutrients on the molar ratios of (**a**) phytate:zinc and (**b**) phytate:iron in grains of the ninety-three wheat accessions. Note: The boxes boundaries indicate the 75% and 25% quartiles; whiskers indicate the 95% confidence limits; the black circles indicate outliers; the solid horizontal line and red circle indicate median and mean, respectively. ∗ ∗∗ is statistically significant at *P* < 0.001, ns indicate not significant at *P* < 0.05.

The molar ratio of PA:Zn was significantly decreased by foliar application of zinc(Fig. 7a), and foliar iron spray did not have a significantly impact on PA:Fe molar ratio (Fig. 7b). Averaged across genotypes, foliar zinc application reduced PA:Zn molar ratio by 17.8% (from 22.3 to 18.3), and foliar iron application reduced PA:Fe molar ratio by 7.9% (from 22.4 to 20.7). The results suggest that foliar zinc application is highly effective in decreasing the PA:Zn molar ratio, which is more efficient than foliar iron application in reducing the PA:Fe molar ratio in wheat grains.

**Figure 8:**
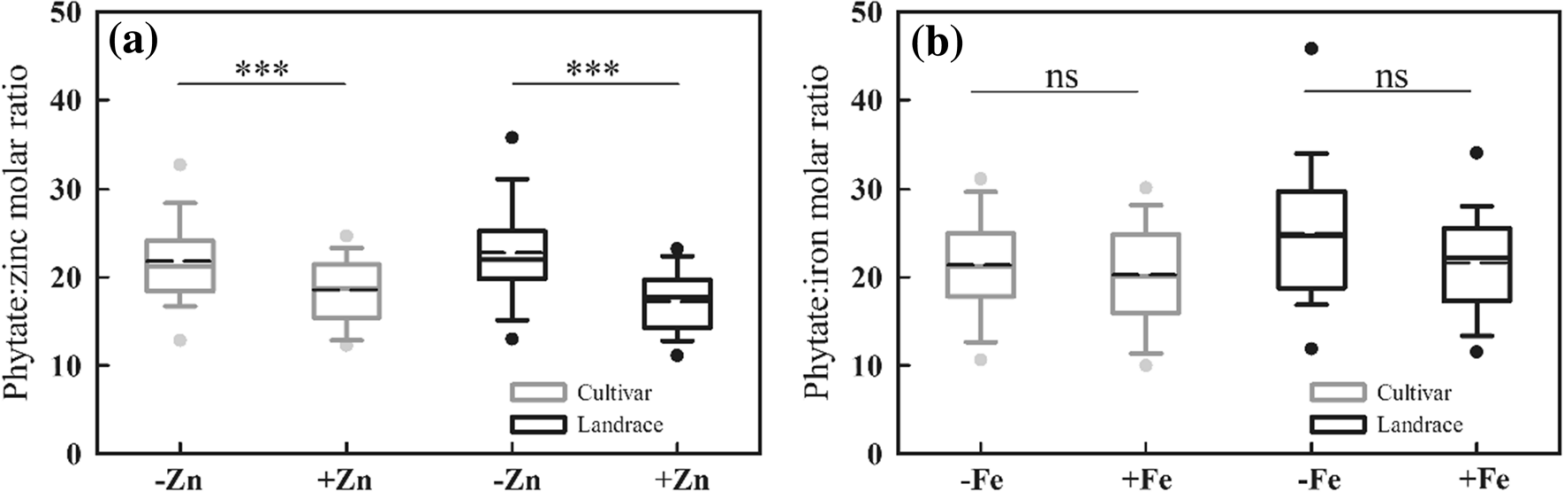
Effects of foliar application of micronutrients on the molar ratios of (**a**) phytate:zinc and (**b**) phytate:iron in grains of Chinese wheat landraces (n = 28) and cultivars (n = 65). Note: The box boundaries indicate upper and lower quartiles; the whiskers indicate the 90th and 10th percentiles; the circles indicate the 95th and 5th percentiles; the solid and dashed horizontal lines indicate median and mean, respectively. ∗ ∗∗ is statistically significant at *P* < 0.001, ns indicate not significant at *P* < 0.05.

The PA:Zn molar ratio in grains in cultivars and landraces were both significantly reduced by foliar zinc application, and the relative reduction in the PA:Zn molar ratio in landrace was 24.0% (from 22.9 to 17.4), which was more than the reduction of 14.9% (from 22.0 to 18.7) in cultivars (Fig. 8a). The results indicate that, with foliar zinc application, landraces had more decrease in the PA:Zn molar ratio as compared to cultivars. The PA:Fe molar ratios in landraces and cultivars were both reduced by foliar iron application, but these reductions were not statistically significant (Fig. 8b) and the relative reduction in the PA:Fe molar ratios in landraces and cultivars were 13.4% and 5.2%, respectively.

## Discussion

Foliar zinc spray is considered as an effective strategy to enhance zinc concentration in wheat grains^7,20^. It has been reported that grain zinc concentration could be increased by 10% to 85% with foliar application of zinc in field experiments^7,15,26^. Our results showed that foliar spray of zinc increase grain zinc concentration by 18.9% (averaged across all genotypes). The different increases in grain zinc concentration may be due to different soil type, genotype, and timing of foliar zinc fertilizer. For example, for wheat grown under field conditions with zinc deficient soils, foliar application of zinc may results in an about twofold increase in grain zinc concentration^9^, and in the same soil type, a up to threefold increase in grain zinc concentration in wheat was reported with a combination of foliar and soil zinc application^19^. The timing of foliar zinc spray is an important factor in increasing grain zinc concentration and field experiment showed that later application of zinc (stem elongation and booting stages) could increase grain zinc concentration by 43% as compared to the earlier application (heading and early milk stages)^24^.

The effectiveness of foliar applied zinc fertilizers in enhancing grain zinc concentration in wheat might relate to the form of zinc. ZnSO_4_ and Zn EDTA are common used when apply foliar zinc fertilizer to wheat^20^. In terms of improving grain zinc concentration in wheat, ZnSO_4_ and Zn EDTA are similarly effective, while in terms of their cost, ZnSO_4_ is much lower than Zn EDTA^20^. So ZnSO_4_ is considered as the most cost-effective foliar zinc fertilizer in wheat production. Previous study reported that foliar application of zinc used the solution mixed with surfactant might improve zinc uptake in wheat plant, however, when surfactant was added to foliar sprays containing ZnSO_4_, which caused negative growth effects^31^.

In this study, PA:Zn molar ratio in grains but not phytic acid concentration were significantly affected by foliar zinc application, and the respective decreases in phytic acid and PA:Zn molar ratio were 1.9% and 17.8%. In Turkey, it has been reported that foliar zinc application reduced phytic acid and PA:Zn molar ratio by 15.5% and 63.9%, respectively^19^. Such a discrepancy between our and their results most likely due to different experimental conditions, in their field experiment, wheat were grown on a severe Zn-deficient soil, and foliar zinc spray resulted in a more than two-fold increase in grain zinc concentration, which is much higher than that in this study (18.9%), so the different increase in grain zinc concentration should accounted for a greater proportion of the variation in PA:Zn molar ratio in these two studies.

Field studies conducted in China and Turkey indicated that, with foliar iron spray, iron concentration in wheat grains could be increased by 28%^25^ and 10%^27^, respectively. However, in this study and another study^23^, foliar iron spray did not have a significantly impact on grain iron concentration in wheat. The results from the above-mentioned studies were in accordance with those reported by Aciksoz et al. (2011)^27^, that the effectiveness of foliar iron spray in improving iron concentration in wheat grains remained controversial. In this study, grain iron increase was less efficient than grain zinc increase by foliar application of micronutrients. These results might be explained by: (i) foliar-applied iron showed poor penetration into leaf tissue and limited mobility in phloem which might limit the transport of iron to developing grain^22,23^; (ii) the chemical form of zinc and iron might be transformed after foliar spray, which might affect the absorption and transport of zinc and iron. For example, it has been reported that the form of foliar-applied zinc phosphite was transformed into a new form after foliar spray, which showed higher diffusion coefficient and may have contributed to higher absorption-transport rate of foliar-applied zinc phosphite^32^; (iii) foliar iron application might have improved iron level in the plant cytoplasm^33^, however, to avoid toxicity, most of the iron was buffered with ferritin or stored as precipitated Fe in multiple intracellular compartments as well as extracellular spaces^34,35^, so only a small portion of iron could be retranslocated to grain^36–38^. Since foliar iron fertilizer might less effective at increasing grain iron concentration, many studies have indicated that, to enhance grain iron concentration in cereals to help alleviate human iron malnutrition, integrated agricultural strategies (e.g., plant breeding, genetic modification and agronomic practices) should be applied^20,22,23^.

In Ethiopia, it has been reported that, for wheat grown in zinc deficient soil, foliar application of zinc increased grain zinc concentration of landrace and cultivar by respectively 82.5% and 45.4%^39^, which is in agreement with our results. This study showed that the grain zinc concentration of cultivars and landraces were both significantly increased by foliar application of zinc, and the increase of grain zinc concentration was greater for landraces (12.5 mg kg^−1^) than that for cultivars (6.6 mg kg^−1^). The different increase of grain zinc concentration between cultivars and landraces can be partially explained by higher grain yield for cultivars compared to landraces (Fig. 6a), which may lead to a strong dilution effect for grain zinc. Furthermore, this study showed that landraces accumulated more zinc in grains than cultivars with zinc application (Fig. 6b), probably because grain zinc in wheat landraces were more source-limited and thus more positively responsive to Zn supply compared to cultivars, since previous study has reported that zinc in wheat grains is usually source-limited^40^. This study also showed that the PA:Zn molar ratios in grains of cultivars and landraces were both significantly reduced by foliar zinc application, and the relative reduction in the PA:Zn molar ratio in landrace was 24.2%, which was more than the reduction of 14.9% in cultivars. This indicates that, with foliar application of zinc, landraces showed more increase in both concentration and bioavailability of zinc in grains as compared with cultivars. However, our results showed that grain iron concentration of both landraces and cultivars do not significantly affected by foliar application of iron. Similar results were reported in a previous study^32^.

## Conclusions

A different response to foliar application of zinc was identified between wheat cultivars and landraces for the concentration and bioavailability of zinc in grains. With foliar application of zinc, wheat landraces had almost two-fold more increase in grain zinc concentration and also greater improvement in zinc bioavailability as compared to cultivars. But foliar iron spray did not significantly affect the concentration and bioavailability of iron in grains of either cultivars or landraces.

## Supplementary Information

Supplementary Information.
