# Supplementary material for: Effects of foliar application of micronutrients on concentration and bioavailability of zinc and iron in wheat landraces and cultivars
Source: Sci Rep. 2021 Nov 23;11:22782. doi: 10.1038/s41598-021-02088-3 (PMC8611096; doi:10.1038/s41598-021-02088-3)
Supplement: Supplementary file 1 — Supplementary Information. [file 41598_2021_2088_MOESM1_ESM.docx]

**Effects of foliar application of micronutrients on concentration and bioavailability of zinc and iron in wheat landraces and cultivars**

**Baozhen Hao ^1^ · Jingli Ma ^1^ · Lina Jiang ^2^ · Xiaojie Wang ^1^ · Yongqu Bai ^1^ · Chuangchuang Zhou ^1^ · Simin Ren ^1^ · Chunxi Li ^2^ · Zhimin Wang ^3^**

^1^ Xinxiang University, School of Life Science and Basic Medicine, Xinxiang 453003, Henan, China

^2^ Henan Normal University, College of Life Sciences, Xinxiang 453007, Henan, China

^3^  China Agricultural University, College of Agronomy, Beijing 100193, China

Table S1 Wheat genotypes used in this study

| **Wheat line name** | **Status** | **Growth habit** | **Wheat line name** | **Status** | **Growth habit** |
| --- | --- | --- | --- | --- | --- |
| Orofen | Cultivar | Spring | Gaojiasuo | Cultivar | Spring |
| Qianjiaomai | Cultivar | Winter | Zhengyin 4 | Cultivar | Spring |
| Triumph | Cultivar | Spring | Gansu 96 | Cultivar | Spring |
| Neixiang 5 | Cultivar | Winter | Shijiazhuang 54 | Cultivar | Winter |
| Ganmai 8 | Cultivar | Winter | Taishan 1 | Cultivar | Winter |
| Huining 10 | Cultivar | Spring | Jinan 2 | Cultivar | Winter |
| Guinong 10 | Cultivar | Winter | Bainong 3217 | Cultivar | Spring |
| Fengmai 11 | Cultivar | Winter | Shijiazhuang 407 | Cultivar | Winter |
| Shuiyuan 86 | Cultivar | Winter | Jimai 2 | Cultivar | Winter |
| Lovrin 10 | Cultivar | Spring | Zhengzhou 6 | Cultivar | Spring |
| Youbao | Cultivar | Winter | Shannong 7859 | Cultivar | Winter |
| Shuwan 8 | Cultivar | Winter | Aimengniu | Cultivar | Winter |
| Bimai26 | Cultivar | Winter | Yumai 49 | Cultivar | Winter |
| Emai6 | Cultivar | Winter | Zhengzhou 741 | Cultivar | Spring |
| Pingyang 27 | Cultivar | Spring | Ningchun 4 | Cultivar | Spring |
| Beijing 8 | Cultivar | Spring | Jinmai 4 | Cultivar | Winter |
| Huadong 6 | Cultivar | Winter | Fan 6 | Cultivar | Spring |
| Sumai 3 | Cultivar | Winter | Yunmai 34 | Cultivar | Winter |
| Anhui 3 | Cultivar | Spring | Pingyuan 50 | Landrace | Winter |
| Taizhong 23 | Cultivar | Spring | Meiqianwu | Landrace | Winter |
| Kashi 1 | Cultivar | Winter | Laoqimai | Landrace | Winter |
| Kashibaipi | Cultivar | Winter | Hanzhongbai | Landrace | Winter |
| Dongfanghong 3 | Cultivar | Winter | Huangguaxian | Landrace | Winter |
| Xinshuguang 1 | Cultivar | Winter | Youmangsaogudan | Landrace | Winter |
| Xinshuguang 6 | Cultivar | Winter | Shengen | Landrace | Spring |
| Nanda 2419 | Cultivar | Winter | Youzimai | Landrace | Winter |
| Early Premium | Cultivar | Winter | Chushanbao | Landrace | Winter |
| Yannong 15 | Cultivar | Spring | Wumangchunmai | Landrace | Winter |
| Zhengmai 9023 | Cultivar | Winter | Huoliaomai | Landrace | Spring |
| Yanzhan 1 | Cultivar | Spring | Jiangdongmen | Landrace | Winter |
| Fengkang 2 | Cultivar | Spring | Daqingmang | Landrace | Winter |
| Bima 1 | Cultivar | Spring | Dabaimai | Landrace | Winter |
| Bima 4 | Cultivar | Spring | Naolaohan | Landrace | Spring |
| Fengchan 3 | Cultivar | Winter | Shanmai | Landrace | Winter |
| Xinong 6028 | Cultivar | Winter | Baiqitou | Landrace | Spring |
| Xiaoyan 6 | Cultivar | Spring | Yangmai | Landrace | Winter |
| Aifeng 3 | Cultivar | Winter | Chejianzi | Landrace | Winter |
| Huzhuhong | Cultivar | Spring | Rumai | Landrace | Spring |
| Changzhi 6406 | Cultivar | Winter | Sankecun | Landrace | Winter |
| Yanan 11 | Cultivar | Winter | Tumangmai | Landrace | Winter |
| Nongda139 | Cultivar | Winter | Hongmangzi | Landrace | Winter |
| Mingxian 169 | Cultivar | Winter | Geerhongmai | Landrace | Spring |
| Yangmai 158 | Cultivar | Winter | Hongheshangtou | Landrace | Winter |
| Zhemai 1 | Cultivar | Spring | Baimaizi | Landrace | Winter |
| Xinkehan 9 | Cultivar | Winter | Jiangmai | Landrace | Winter |
| Kefeng 3 | Cultivar | Winter | Hongxumai | Landrace | Winter |
| Funo | Cultivar | Spring |  |  |  |

Table S2 Variance components and broad sense heritability for grain zinc and iron concentrations in 93 wheat accessions evaluated at two environments

| **Variance component** | **All wheat accessions** | | **Cultivars** | | **Landraces** | |
| --- | --- | --- | --- | --- | --- | --- |
|  | **Zn** | **Fe** | **Zn** | **Fe** | **Zn** | **Fe** |
| σ^2^_G_ | 24.27 | 13.75 | 13.54 | 7.20 | 37.02 | 33.61 |
| σ^2^_GE_ | 53.75 | 79.60 | 49.06 | 82.28 | 64.82 | 64.99 |
| σ^2^_R_ | 6.52 | 9.89 | 7.38 | 10.01 | 4.56 | 9.79 |
| Broad sense heritability (%) | 46 | 25 | 34 | 14 | 53 | 50 |

σ^2^_G_ is the genotypic variance, σ^2^_GE_ is the genotype × environment variance, and σ^2^_R_ is the residual error variance for r replicates and y years.

Table S3 Data collected for Experiment I

| **Wheat line name** | **2017/18 season** | | | **2018/19 season** | | |
| --- | --- | --- | --- | --- | --- | --- |
|  | **Grain Zn concentration (mg kg^-1^)** | **Grain Fe concentration (mg kg^-1^)** | **Grain yield (Mg ha^-1^)** | **Grain Zn concentration (mg kg^-1^)** | **Grain Fe concentration (mg kg^-1^)** | **Grain yield (Mg ha^-1^)** |
| Orofen | 29.48 | 32.25 | 7.50 | 37.26 | 41.09 | 4.73 |
| Qianjiaomai | 27.46 | 27.29 | 6.22 | 32.60 | 35.13 | 5.87 |
| Triumph | 30.52 | 30.85 | 4.55 | 54.26 | 40.82 | 2.85 |
| Neixiang 5 | 31.56 | 34.17 | 7.40 | 41.19 | 26.90 | 7.87 |
| Ganmai 8 | 25.79 | 32.67 | 2.96 | 41.35 | 40.78 | 2.03 |
| Huining 10 | 30.26 | 28.84 | 5.01 | 34.83 | 42.24 | 5.54 |
| Guinong 10 | 30.05 | 32.77 | 5.00 | 46.99 | 47.33 | 4.65 |
| Fengmai 11 | 29.97 | 34.22 | 7.31 | 37.84 | 29.86 | 8.40 |
| Shuiyuan 86 | 28.50 | 36.50 | 3.15 | 33.71 | 31.92 | 3.55 |
| Lovrin 10 | 31.49 | 36.61 | 7.03 | 38.97 | 36.14 | 8.24 |
| Youbao | 31.92 | 36.86 | 3.05 | 41.44 | 40.12 | 2.16 |
| Shuwan 8 | 27.96 | 37.75 | 6.82 | 37.49 | 45.75 | 5.21 |
| Bimai26 | 28.44 | 35.83 | 5.82 | 29.48 | 46.16 | 3.41 |
| Emai6 | 46.77 | 46.75 | 7.30 | 37.49 | 40.05 | 7.44 |
| Pingyang 27 | 54.42 | 51.06 | 4.22 | 70.15 | 37.57 | 2.58 |
| Beijing 8 | 45.90 | 40.50 | 8.17 | 53.20 | 62.85 | 5.43 |
| Huadong 6 | 55.07 | 37.33 | 5.34 | 47.68 | 41.51 | 2.99 |
| Sumai 3 | 54.16 | 39.23 | 8.07 | 40.39 | 38.24 | 7.62 |
| Anhui 3 | 44.40 | 41.89 | 4.89 | 43.99 | 42.29 | 5.57 |
| Taizhong 23 | 44.67 | 43.54 | 3.00 | 74.12 | 60.96 | 3.22 |
| Kashi 1 | 44.83 | 43.70 | 4.65 | 38.71 | 33.56 | 3.24 |
| Kashibaipi | 44.68 | 43.77 | 1.59 | 31.16 | 26.20 | 1.76 |
| Dongfanghong 3 | 32.51 | 32.64 | 7.25 | 79.26 | 57.41 | 6.39 |
| Xinshuguang 1 | 43.68 | 34.56 | 4.58 | 40.66 | 32.04 | 4.64 |
| Xinshuguang 6 | 40.63 | 32.45 | 8.36 | 32.05 | 24.97 | 8.42 |
| Nanda 2419 | 36.24 | 33.68 | 7.23 | 37.35 | 25.89 | 7.51 |
| Early Premium | 36.35 | 33.59 | 7.07 | 49.98 | 43.18 | 4.46 |
| Yannong 15 | 32.23 | 34.21 | 4.36 | 35.04 | 21.50 | 2.84 |
| Zhengmai 9023 | 32.64 | 34.87 | 11.41 | 37.83 | 40.94 | 9.03 |
| Yanzhan 1 | 35.63 | 34.38 | 5.80 | 38.05 | 30.43 | 7.64 |
| Fengkang 2 | 38.18 | 48.08 | 4.81 | 38.71 | 60.04 | 4.85 |
| Bima 1 | 35.55 | 45.05 | 6.00 | 52.24 | 38.45 | 6.34 |
| Bima 4 | 33.10 | 45.72 | 6.56 | 47.89 | 42.16 | 5.65 |
| Fengchan 3 | 35.88 | 45.64 | 10.38 | 51.97 | 37.00 | 9.16 |
| Xinong 6028 | 35.54 | 46.63 | 1.80 | 51.08 | 36.08 | 1.72 |
| Xiaoyan 6 | 38.42 | 49.30 | 7.84 | 44.92 | 37.49 | 5.81 |
| Aifeng 3 | 39.68 | 45.57 | 5.28 | 39.29 | 29.53 | 3.31 |
| Huzhuhong | 35.75 | 49.05 | 4.75 | 30.89 | 36.44 | 5.40 |
| Changzhi 6406 | 39.41 | 41.69 | 8.63 | 40.51 | 83.18 | 5.07 |
| Yanan 11 | 40.08 | 38.58 | 8.88 | 46.70 | 78.81 | 5.56 |
| Nongda139 | 40.96 | 42.76 | 5.96 | 44.33 | 75.32 | 6.97 |
| Mingxian 169 | 36.70 | 38.11 | 4.57 | 51.29 | 52.21 | 4.66 |
| Yangmai 158 | 43.21 | 43.25 | 6.12 | 28.34 | 35.03 | 6.36 |
| Zhemai 1 | 41.61 | 40.76 | 4.67 | 57.38 | 57.97 | 4.55 |
| Xinkehan 9 | 36.86 | 35.57 | 5.74 | 49.18 | 46.98 | 6.40 |
| Kefeng 3 | 43.44 | 35.64 | 3.98 | 43.43 | 32.87 | 4.12 |
| Funo | 36.76 | 42.45 | 6.31 | 41.66 | 30.17 | 7.08 |
| Gaojiasuo | 35.19 | 37.53 | 4.73 | 37.44 | 31.94 | 3.86 |
| Zhengyin 4 | 35.50 | 38.54 | 4.01 | 39.24 | 31.73 | 4.55 |
| Gansu 96 | 38.53 | 36.14 | 5.53 | 49.31 | 36.98 | 5.07 |
| Shijiazhuang 54 | 34.10 | 42.75 | 4.88 | 38.52 | 33.80 | 5.52 |
| Taishan 1 | 38.06 | 36.72 | 5.47 | 35.21 | 23.05 | 5.11 |
| Jinan 2 | 36.15 | 44.72 | 3.48 | 35.54 | 26.75 | 3.53 |
| Bainong 3217 | 42.56 | 42.11 | 2.77 | 49.14 | 31.51 | 1.74 |
| Shijiazhuang 407 | 32.04 | 38.45 | 6.99 | 47.04 | 31.52 | 5.13 |
| Jimai 2 | 35.61 | 35.74 | 8.66 | 46.17 | 28.32 | 8.56 |
| Zhengzhou 6 | 32.14 | 36.53 | 6.78 | 50.14 | 44.38 | 6.10 |
| Shannong 7859 | 36.13 | 44.09 | 7.27 | 46.98 | 34.23 | 7.05 |
| Aimengniu | 40.78 | 42.27 | 9.23 | 42.96 | 32.36 | 8.43 |
| Yumai 49 | 39.08 | 38.23 | 4.80 | 36.77 | 30.08 | 4.25 |
| Zhengzhou 741 | 37.22 | 41.55 | 3.67 | 43.29 | 35.00 | 2.61 |
| Ningchun 4 | 37.52 | 36.62 | 3.46 | 39.41 | 37.10 | 2.71 |
| Jinmai 4 | 43.91 | 40.35 | 4.36 | 42.13 | 37.82 | 4.48 |
| Fan 6 | 32.87 | 41.88 | 6.50 | 42.86 | 50.32 | 7.50 |
| Yunmai 34 | 35.57 | 35.35 | 5.76 | 45.47 | 37.34 | 4.35 |
| Pingyuan 50 | 31.33 | 33.52 | 7.03 | 38.08 | 29.65 | 7.57 |
| Meiqianwu | 25.22 | 34.18 | 5.15 | 33.50 | 47.15 | 5.71 |
| Laoqimai | 29.70 | 34.81 | 2.01 | 37.80 | 23.35 | 1.61 |
| Hanzhongbai | 31.12 | 31.77 | 4.29 | 44.03 | 32.66 | 4.25 |
| Huangguaxian | 31.06 | 51.18 | 2.74 | 45.97 | 34.16 | 3.08 |
| Youmangsaogudan | 31.80 | 50.22 | 3.96 | 50.54 | 47.14 | 3.65 |
| Shengen | 30.45 | 38.99 | 5.08 | 51.86 | 33.65 | 6.24 |
| Youzimai | 31.15 | 37.56 | 6.66 | 43.23 | 25.94 | 5.50 |
| Chushanbao | 31.64 | 41.93 | 4.06 | 46.67 | 39.84 | 3.27 |
| Wumangchunmai | 61.79 | 32.37 | 1.79 | 44.38 | 34.85 | 2.43 |
| Huoliaomai | 54.89 | 48.84 | 1.63 | 71.61 | 85.12 | 1.26 |
| Jiangdongmen | 60.95 | 51.49 | 7.29 | 56.09 | 47.78 | 7.38 |
| Daqingmang | 53.17 | 57.10 | 4.96 | 48.93 | 51.14 | 5.18 |
| Dabaimai | 54.52 | 54.41 | 5.02 | 42.29 | 36.42 | 6.76 |
| Naolaohan | 52.67 | 58.20 | 1.85 | 41.68 | 30.75 | 1.77 |
| Shanmai | 52.73 | 54.99 | 1.09 | 59.59 | 38.78 | 1.28 |
| Baiqitou | 51.86 | 53.04 | 3.22 | 44.00 | 33.44 | 2.34 |
| Yangmai | 67.25 | 52.03 | 2.26 | 52.50 | 41.56 | 2.54 |
| Chejianzi | 54.63 | 44.20 | 3.96 | 50.00 | 40.23 | 1.68 |
| Rumai | 44.92 | 36.44 | 8.21 | 42.34 | 41.38 | 8.98 |
| Sankecun | 54.41 | 41.66 | 3.68 | 39.72 | 44.59 | 4.83 |
| Tumangmai | 32.49 | 30.71 | 4.61 | 37.63 | 30.05 | 5.37 |
| Hongmangzi | 36.49 | 33.77 | 1.94 | 40.90 | 27.83 | 2.24 |
| Geerhongmai | 42.79 | 45.56 | 2.43 | 43.39 | 37.98 | 2.54 |
| Hongheshangtou | 41.58 | 39.62 | 3.38 | 49.30 | 30.74 | 2.76 |
| Baimaizi | 37.77 | 43.88 | 3.94 | 48.04 | 34.15 | 3.86 |
| Jiangmai | 32.37 | 38.12 | 2.96 | 54.83 | 40.50 | 3.27 |
| Hongxumai | 42.08 | 44.89 | 2.26 | 48.78 | 39.93 | 2.36 |

Table S4 Data collected for Experiment II

| **Wheat line name** | **No zinc fertilizer** | | | | **With zinc fertilizer** | | | |  |
| --- | --- | --- | --- | --- | --- | --- | --- | --- | --- |
|  | **Grain Zn concentration (mg kg^-1^)** | **Phytate concentration (mg g^-1^)** | **Phytate:Zinc molar ratio** | **Grain yield**  **(Mg ha^-1^)** | **Grain Zn concentration (mg kg^-1^)** | **Phytate concentration (mg g^-1^)** | **Phytate:Zinc molar ratio** | **Grain yield**  **(Mg ha^-1^)** |  |
| Orofen | 37.26 | 8.65 | 23.02 | 4.73 | 38.78 | 9.78 | 24.98 | 4.66 | |
| Qianjiaomai | 32.60 | 12.82 | 38.96 | 5.87 | 43.61 | 7.75 | 17.61 | 5.98 | |
| Triumph | 54.26 | 8.28 | 15.12 | 2.85 | 53.08 | 7.85 | 14.66 | 3.11 | |
| Neixiang 5 | 41.19 | 8.67 | 20.85 | 7.87 | 44.63 | 9.25 | 20.54 | 7.52 | |
| Ganmai 8 | 41.35 | 10.19 | 24.41 | 2.03 | 42.51 | 9.34 | 21.77 | 2.24 | |
| Huining 10 | 34.83 | 9.87 | 28.07 | 5.54 | 37.10 | 9.65 | 25.78 | 4.39 | |
| Guinong 10 | 46.99 | 10.09 | 21.28 | 4.65 | 44.18 | 9.30 | 20.86 | 4.11 | |
| Fengmai 11 | 37.84 | 10.25 | 26.83 | 8.40 | 55.39 | 8.73 | 15.62 | 7.05 | |
| Shuiyuan 86 | 33.71 | 9.51 | 27.94 | 3.55 | 27.57 | 10.22 | 36.74 | 4.08 | |
| Lovrin 10 | 38.97 | 8.50 | 21.62 | 8.24 | 42.31 | 7.38 | 17.27 | 9.18 | |
| Youbao | 41.44 | 9.08 | 21.71 | 2.16 | 40.52 | 9.33 | 22.81 | 1.81 | |
| Shuwan 8 | 37.49 | 9.30 | 24.59 | 5.21 | 39.30 | 9.73 | 24.54 | 5.50 | |
| Bimai26 | 29.48 | 9.57 | 32.15 | 3.41 | 45.54 | 9.51 | 20.70 | 4.14 | |
| Emai6 | 37.49 | 7.78 | 20.56 | 7.44 | 45.92 | 7.96 | 17.19 | 8.66 | |
| Pingyang 27 | 70.15 | 8.47 | 11.97 | 2.58 | 51.53 | 9.18 | 17.65 | 2.59 | |
| Beijing 8 | 53.20 | 8.68 | 16.17 | 5.43 | 64.81 | 7.68 | 11.74 | 6.22 | |
| Huadong 6 | 47.68 | 8.55 | 17.77 | 2.99 | 62.52 | 7.84 | 12.43 | 3.50 | |
| Sumai 3 | 40.39 | 8.22 | 20.16 | 7.62 | 52.20 | 8.37 | 15.88 | 8.09 | |
| Anhui 3 | 43.99 | 9.07 | 20.42 | 5.57 | 39.17 | 8.62 | 21.80 | 4.87 | |
| Taizhong 23 | 74.12 | 7.89 | 10.54 | 3.22 | 49.51 | 8.18 | 16.37 | 3.88 | |
| Kashi 1 | 38.71 | 9.47 | 24.25 | 3.24 | 53.89 | 10.76 | 19.78 | 2.80 | |
| Kashibaipi | 31.16 | 9.24 | 29.40 | 1.76 | 55.02 | 9.71 | 17.49 | 2.26 | |
| Dongfanghong 3 | 79.26 | 9.35 | 11.69 | 6.39 | 67.80 | 9.74 | 14.23 | 7.46 | |
| Xinshuguang 1 | 40.66 | 9.42 | 22.94 | 4.64 | 43.28 | 8.74 | 20.00 | 5.50 | |
| Xinshuguang 6 | 32.05 | 8.96 | 27.69 | 8.42 | 43.19 | 8.28 | 19.00 | 8.64 | |
| Nanda 2419 | 37.35 | 8.25 | 21.89 | 7.51 | 39.04 | 9.53 | 24.20 | 7.76 | |
| Early Premium | 49.98 | 9.09 | 18.02 | 4.46 | 49.41 | 8.05 | 16.14 | 4.63 | |
| Yannong 15 | 35.04 | 11.78 | 33.29 | 2.84 | 53.67 | 10.77 | 19.89 | 3.02 | |
| Zhengmai 9023 | 37.83 | 9.00 | 23.57 | 9.03 | 46.28 | 10.34 | 22.14 | 7.93 | |
| Yanzhan 1 | 38.05 | 9.86 | 25.67 | 7.64 | 43.99 | 10.26 | 23.12 | 4.38 | |
| Fengkang 2 | 38.71 | 8.38 | 21.44 | 4.85 | 62.13 | 7.96 | 12.70 | 5.51 | |
| Bima 1 | 52.24 | 8.84 | 16.77 | 6.34 | 60.66 | 7.73 | 12.63 | 5.80 | |
| Bima 4 | 47.89 | 8.53 | 17.65 | 5.65 | 53.79 | 8.21 | 15.12 | 6.29 | |
| Fengchan 3 | 51.97 | 8.85 | 16.87 | 9.16 | 62.78 | 9.40 | 14.83 | 5.78 | |
| Xinong 6028 | 51.08 | 9.25 | 17.95 | 1.72 | 52.04 | 11.13 | 21.19 | 1.58 | |
| Xiaoyan 6 | 44.92 | 8.67 | 19.12 | 5.81 | 61.88 | 9.26 | 14.82 | 7.15 | |
| Aifeng 3 | 39.29 | 8.72 | 22.00 | 3.31 | 46.66 | 9.39 | 19.95 | 2.23 | |
| Huzhuhong | 30.89 | 10.68 | 34.24 | 5.40 | 49.77 | 11.04 | 21.99 | 5.88 | |
| Changzhi 6406 | 40.51 | 8.85 | 21.65 | 5.07 | 60.05 | 7.97 | 13.16 | 4.69 | |
| Yanan 11 | 46.70 | 8.82 | 18.71 | 5.56 | 71.05 | 8.45 | 11.79 | 5.38 | |
| Nongda139 | 44.33 | 9.10 | 20.35 | 6.97 | 61.85 | 8.63 | 13.83 | 6.76 | |
| Mingxian 169 | 51.29 | 8.87 | 17.13 | 4.66 | 68.92 | 8.56 | 12.31 | 4.89 | |
| Yangmai 158 | 28.34 | 9.13 | 31.91 | 6.36 | 44.63 | 7.31 | 16.23 | 7.93 | |
| Zhemai 1 | 57.38 | 11.35 | 19.60 | 4.55 | 59.76 | 11.17 | 18.52 | 4.84 | |
| Xinkehan 9 | 49.18 | 8.75 | 17.63 | 6.40 | 44.31 | 8.94 | 19.99 | 5.95 | |
| Kefeng 3 | 43.43 | 8.65 | 19.72 | 4.12 | 51.79 | 8.38 | 16.03 | 3.95 | |
| Funo | 41.66 | 8.27 | 19.66 | 7.08 | 38.24 | 8.33 | 21.57 | 5.40 | |
| Gaojiasuo | 37.44 | 8.69 | 23.00 | 3.86 | 39.64 | 8.94 | 22.35 | 4.85 | |
| Zhengyin 4 | 39.24 | 8.90 | 22.47 | 4.55 | 39.92 | 8.73 | 21.66 | 4.20 | |
| Gansu 96 | 49.31 | 8.73 | 17.53 | 5.07 | 65.37 | 8.65 | 13.12 | 5.87 | |
| Shijiazhuang 54 | 38.52 | 8.74 | 22.48 | 5.52 | 49.71 | 8.21 | 16.36 | 4.51 | |
| Taishan 1 | 35.21 | 8.51 | 23.94 | 5.11 | 41.81 | 8.48 | 20.10 | 6.48 | |
| Jinan 2 | 35.54 | 9.53 | 26.56 | 3.53 | 39.81 | 9.12 | 22.70 | 4.02 | |
| Bainong 3217 | 49.14 | 9.15 | 18.45 | 1.74 | 52.07 | 8.94 | 17.01 | 2.27 | |
| Shijiazhuang 407 | 47.04 | 8.95 | 18.86 | 5.13 | 54.13 | 9.64 | 17.65 | 5.31 | |
| Jimai 2 | 46.17 | 8.80 | 18.89 | 8.56 | 50.31 | 8.82 | 17.37 | 9.28 | |
| Zhengzhou 6 | 50.14 | 8.75 | 17.28 | 6.10 | 42.06 | 9.91 | 23.33 | 5.35 | |
| Shannong 7859 | 46.98 | 9.37 | 19.77 | 7.05 | 65.31 | 9.74 | 14.78 | 9.60 | |
| Aimengniu | 42.96 | 9.38 | 21.64 | 8.43 | 46.41 | 8.82 | 18.82 | 9.27 | |
| Yumai 49 | 36.77 | 10.21 | 27.52 | 4.25 | 46.31 | 11.08 | 23.70 | 5.93 | |
| Zhengzhou 741 | 43.29 | 10.38 | 23.75 | 2.61 | 64.86 | 10.14 | 15.49 | 3.28 | |
| Ningchun 4 | 39.41 | 10.18 | 25.59 | 2.71 | 45.78 | 9.68 | 20.96 | 2.50 | |
| Jinmai 4 | 42.13 | 9.74 | 22.90 | 4.48 | 50.92 | 10.11 | 19.67 | 5.49 | |
| Fan 6 | 42.86 | 9.25 | 21.38 | 7.50 | 45.57 | 8.91 | 19.37 | 8.94 | |
| Yunmai 34 | 45.47 | 9.81 | 21.38 | 4.35 | 46.33 | 9.75 | 20.85 | 4.42 | |
| Pingyuan 50 | 38.08 | 11.24 | 29.24 | 7.57 | 49.47 | 11.31 | 22.66 | 8.20 | |
| Meiqianwu | 33.50 | 10.58 | 31.28 | 5.71 | 59.25 | 11.04 | 18.47 | 5.69 | |
| Laoqimai | 37.80 | 15.01 | 39.35 | 1.61 | 47.34 | 11.45 | 23.96 | 1.46 | |
| Hanzhongbai | 44.03 | 9.71 | 21.86 | 4.25 | 49.36 | 9.63 | 19.32 | 4.62 | |
| Huangguaxian | 45.97 | 11.02 | 23.76 | 3.08 | 71.07 | 10.05 | 14.02 | 3.97 | |
| Youmangsaogudan | 50.54 | 10.51 | 20.60 | 3.65 | 74.10 | 10.51 | 14.05 | 3.56 | |
| Shengen | 51.86 | 7.64 | 14.60 | 6.24 | 50.19 | 7.79 | 15.39 | 5.67 | |
| Youzimai | 43.23 | 10.44 | 23.92 | 5.50 | 58.89 | 10.59 | 17.82 | 6.16 | |
| Chushanbao | 46.67 | 11.82 | 25.09 | 3.27 | 51.37 | 10.35 | 19.97 | 4.06 | |
| Wumangchunmai | 44.38 | 9.80 | 21.89 | 2.43 | 57.47 | 10.40 | 17.94 | 3.08 | |
| Huoliaomai | 71.61 | 8.55 | 11.83 | 1.26 | 76.32 | 9.40 | 12.21 | 1.23 | |
| Jiangdongmen | 56.09 | 8.67 | 15.32 | 7.38 | 76.56 | 8.01 | 10.36 | 5.37 | |
| Daqingmang | 48.93 | 11.17 | 22.62 | 5.18 | 63.27 | 9.78 | 15.31 | 6.01 | |
| Dabaimai | 42.29 | 10.88 | 25.50 | 6.76 | 53.77 | 10.66 | 19.63 | 8.49 | |
| Naolaohan | 41.68 | 11.08 | 26.35 | 1.77 | 48.74 | 10.81 | 21.98 | 1.84 | |
| Shanmai | 59.59 | 10.91 | 18.14 | 1.28 | 58.97 | 11.91 | 20.01 | 1.25 | |
| Baiqitou | 44.00 | 9.98 | 22.47 | 2.34 | 71.09 | 9.55 | 13.32 | 2.22 | |
| Yangmai | 52.50 | 11.36 | 21.45 | 2.54 | 57.83 | 10.71 | 18.35 | 2.76 | |
| Chejianzi | 50.00 | 9.05 | 17.93 | 1.68 | 72.33 | 9.47 | 12.97 | 2.00 | |
| Rumai | 42.34 | 8.49 | 19.88 | 8.98 | 51.63 | 8.93 | 17.15 | 8.14 | |
| Sankecun | 39.72 | 8.77 | 21.88 | 4.83 | 65.05 | 8.92 | 13.59 | 4.72 | |
| Tumangmai | 37.63 | 12.15 | 31.99 | 5.37 | 49.96 | 10.39 | 20.60 | 5.74 | |
| Hongmangzi | 40.90 | 9.79 | 23.72 | 2.24 | 58.10 | 10.04 | 17.12 | 1.95 | |
| Geerhongmai | 43.39 | 11.44 | 26.13 | 2.54 | 45.74 | 10.42 | 22.56 | 3.01 | |
| Hongheshangtou | 49.30 | 11.84 | 23.80 | 2.76 | 65.58 | 10.18 | 15.38 | 3.78 | |
| Baimaizi | 48.04 | 10.25 | 21.14 | 3.86 | 56.08 | 10.68 | 18.87 | 4.10 | |
| Jiangmai | 54.83 | 10.51 | 19.00 | 3.27 | 60.48 | 9.67 | 15.84 | 3.51 | |
| Hongxumai | 48.78 | 9.96 | 20.23 | 2.36 | 59.06 | 10.73 | 18.00 | 2.18 | |

Table S5 Data collected for Experiment III

| **Wheat line name** | **No iron fertilizer** | | | | **With iron fertilizer** | | | |  |
| --- | --- | --- | --- | --- | --- | --- | --- | --- | --- |
|  | **Grain Fe concentration (mg kg^-1^)** | **Phytate concentration (mg g^-1^)** | **Phytate:Iron molar ratio** | **Grain yield**  **(Mg ha^-1^)** | **Grain Fe concentration (mg kg^-1^)** | **Phytate concentration (mg g^-1^)** | **Phytate:Iron molar ratio** | **Grain yield**  **(Mg ha^-1^)** |  |
| Orofen | 41.09 | 8.82 | 18.15 | 4.73 | 38.10 | 9.00 | 19.99 | 4.38 | |
| Qianjiaomai | 35.13 | 12.97 | 31.24 | 5.87 | 33.53 | 7.83 | 19.76 | 6.39 | |
| Triumph | 40.82 | 8.04 | 16.67 | 2.85 | 36.45 | 7.70 | 17.87 | 2.72 | |
| Neixiang 5 | 26.90 | 8.69 | 27.33 | 7.87 | 26.76 | 9.56 | 30.23 | 8.48 | |
| Ganmai 8 | 40.78 | 10.66 | 22.12 | 2.03 | 46.69 | 8.84 | 16.02 | 1.89 | |
| Huining 10 | 42.24 | 9.99 | 20.01 | 5.54 | 30.80 | 9.58 | 26.32 | 7.27 | |
| Guinong 10 | 47.33 | 10.14 | 18.14 | 4.65 | 55.33 | 8.82 | 13.49 | 3.81 | |
| Fengmai 11 | 29.86 | 10.14 | 28.74 | 8.40 | 40.40 | 8.42 | 17.63 | 10.66 | |
| Shuiyuan 86 | 31.92 | 10.65 | 28.22 | 3.55 | 29.03 | 10.42 | 30.37 | 3.87 | |
| Lovrin 10 | 36.14 | 8.35 | 19.54 | 8.24 | 39.83 | 7.35 | 15.60 | 7.34 | |
| Youbao | 40.12 | 9.28 | 19.58 | 2.16 | 28.84 | 9.39 | 27.54 | 2.83 | |
| Shuwan 8 | 45.75 | 9.60 | 17.75 | 5.21 | 36.44 | 9.74 | 22.62 | 4.55 | |
| Bimai26 | 46.16 | 10.13 | 18.56 | 3.41 | 59.11 | 9.27 | 13.27 | 3.20 | |
| Emai6 | 40.05 | 7.85 | 16.59 | 7.44 | 42.13 | 7.91 | 15.89 | 7.00 | |
| Pingyang 27 | 37.57 | 8.18 | 18.41 | 2.58 | 42.49 | 9.46 | 18.85 | 2.96 | |
| Beijing 8 | 62.85 | 8.64 | 11.63 | 5.43 | 64.12 | 7.45 | 9.84 | 7.28 | |
| Huadong 6 | 41.51 | 9.13 | 18.62 | 2.99 | 48.94 | 7.75 | 13.41 | 3.19 | |
| Sumai 3 | 38.24 | 8.50 | 18.80 | 7.62 | 32.04 | 8.69 | 22.94 | 8.52 | |
| Anhui 3 | 42.29 | 9.45 | 18.90 | 5.57 | 42.47 | 9.17 | 18.28 | 5.28 | |
| Taizhong 23 | 60.96 | 7.70 | 10.69 | 3.22 | 39.45 | 8.20 | 17.59 | 3.98 | |
| Kashi 1 | 33.56 | 9.22 | 23.26 | 3.24 | 43.38 | 10.88 | 21.22 | 2.54 | |
| Kashibaipi | 26.20 | 9.49 | 30.66 | 1.76 | 43.42 | 9.78 | 19.06 | 1.71 | |
| Dongfanghong 3 | 57.41 | 9.05 | 13.33 | 6.39 | 82.82 | 10.24 | 10.47 | 8.40 | |
| Xinshuguang 1 | 32.04 | 9.41 | 24.84 | 4.64 | 31.01 | 8.75 | 23.87 | 4.97 | |
| Xinshuguang 6 | 24.97 | 9.30 | 31.52 | 8.42 | 23.61 | 8.06 | 28.88 | 7.67 | |
| Nanda 2419 | 25.89 | 7.93 | 25.92 | 7.51 | 28.33 | 9.25 | 27.63 | 8.95 | |
| Early Premium | 43.18 | 9.10 | 17.83 | 4.46 | 32.72 | 8.37 | 21.63 | 4.11 | |
| Yannong 15 | 21.50 | 11.60 | 45.63 | 2.84 | 29.96 | 10.78 | 30.45 | 2.88 | |
| Zhengmai 9023 | 40.94 | 9.38 | 19.38 | 9.03 | 41.21 | 10.20 | 20.94 | 8.82 | |
| Yanzhan 1 | 30.43 | 9.94 | 27.64 | 7.64 | 30.27 | 9.63 | 26.94 | 6.87 | |
| Fengkang 2 | 60.04 | 8.09 | 11.40 | 4.85 | 67.63 | 7.63 | 9.55 | 6.63 | |
| Bima 1 | 38.45 | 8.46 | 18.62 | 6.34 | 40.20 | 7.92 | 16.66 | 5.42 | |
| Bima 4 | 42.16 | 8.44 | 16.93 | 5.65 | 35.53 | 8.26 | 19.67 | 7.23 | |
| Fengchan 3 | 37.00 | 8.63 | 19.72 | 9.16 | 41.75 | 9.64 | 19.54 | 9.19 | |
| Xinong 6028 | 36.08 | 9.47 | 22.22 | 1.72 | 33.00 | 10.71 | 27.46 | 1.80 | |
| Xiaoyan 6 | 37.49 | 9.36 | 21.12 | 5.81 | 37.21 | 8.89 | 20.22 | 6.38 | |
| Aifeng 3 | 29.53 | 8.79 | 25.20 | 3.31 | 29.38 | 9.12 | 26.27 | 2.21 | |
| Huzhuhong | 36.44 | 10.76 | 25.00 | 5.40 | 39.14 | 11.06 | 23.91 | 7.12 | |
| Changzhi 6406 | 83.18 | 8.79 | 8.94 | 5.07 | 66.67 | 8.15 | 10.35 | 4.40 | |
| Yanan 11 | 78.81 | 8.74 | 9.38 | 5.56 | 70.60 | 8.56 | 10.26 | 7.24 | |
| Nongda139 | 75.32 | 9.38 | 10.54 | 6.97 | 77.61 | 8.88 | 9.68 | 8.58 | |
| Mingxian 169 | 52.21 | 8.93 | 14.47 | 4.66 | 60.71 | 8.56 | 11.93 | 4.03 | |
| Yangmai 158 | 35.03 | 9.06 | 21.89 | 6.36 | 46.48 | 7.53 | 13.71 | 7.31 | |
| Zhemai 1 | 57.97 | 11.70 | 17.07 | 4.55 | 53.95 | 11.40 | 17.88 | 5.02 | |
| Xinkehan 9 | 46.98 | 8.61 | 15.52 | 6.40 | 34.11 | 8.72 | 21.63 | 5.63 | |
| Kefeng 3 | 32.87 | 8.47 | 21.82 | 4.12 | 34.57 | 8.42 | 20.62 | 4.53 | |
| Funo | 30.17 | 8.05 | 22.57 | 7.08 | 34.11 | 8.16 | 20.25 | 5.10 | |
| Gaojiasuo | 31.94 | 8.92 | 23.63 | 3.86 | 29.50 | 8.46 | 24.27 | 4.15 | |
| Zhengyin 4 | 31.73 | 9.29 | 24.79 | 4.55 | 29.72 | 8.08 | 23.01 | 4.41 | |
| Gansu 96 | 36.98 | 8.34 | 19.09 | 5.07 | 36.22 | 8.45 | 19.75 | 6.11 | |
| Shijiazhuang 54 | 33.80 | 8.46 | 21.19 | 5.52 | 35.89 | 8.26 | 19.48 | 6.07 | |
| Taishan 1 | 23.05 | 8.22 | 30.19 | 5.11 | 28.40 | 8.51 | 25.35 | 4.70 | |
| Jinan 2 | 26.75 | 9.67 | 30.57 | 3.53 | 26.31 | 9.21 | 29.60 | 3.74 | |
| Bainong 3217 | 31.51 | 8.96 | 24.07 | 1.74 | 30.07 | 9.49 | 26.71 | 1.40 | |
| Shijiazhuang 407 | 31.52 | 9.12 | 24.48 | 5.13 | 34.81 | 9.80 | 23.83 | 7.03 | |
| Jimai 2 | 28.32 | 9.07 | 27.09 | 8.56 | 28.86 | 8.81 | 25.85 | 7.51 | |
| Zhengzhou 6 | 44.38 | 8.49 | 16.19 | 6.10 | 31.48 | 9.56 | 25.71 | 5.27 | |
| Shannong 7859 | 34.23 | 9.44 | 23.33 | 7.05 | 37.19 | 9.72 | 22.11 | 9.00 | |
| Aimengniu | 32.36 | 9.76 | 25.53 | 8.43 | 52.22 | 8.44 | 13.68 | 7.33 | |
| Yumai 49 | 30.08 | 10.40 | 29.25 | 4.25 | 32.27 | 11.21 | 29.40 | 4.41 | |
| Zhengzhou 741 | 35.00 | 9.97 | 24.11 | 2.61 | 40.31 | 9.85 | 20.67 | 3.59 | |
| Ningchun 4 | 37.10 | 9.74 | 22.22 | 2.71 | 43.49 | 9.41 | 18.30 | 2.71 | |
| Jinmai 4 | 37.82 | 9.72 | 21.73 | 4.48 | 44.61 | 10.60 | 20.11 | 4.79 | |
| Fan 6 | 50.32 | 9.47 | 15.92 | 7.50 | 48.71 | 8.55 | 14.86 | 8.33 | |
| Yunmai 34 | 37.34 | 9.72 | 22.02 | 4.35 | 54.37 | 9.75 | 15.18 | 4.20 | |
| Pingyuan 50 | 29.65 | 11.26 | 32.12 | 7.57 | 29.89 | 11.35 | 32.13 | 7.68 | |
| Meiqianwu | 47.15 | 10.77 | 19.33 | 5.71 | 33.39 | 10.51 | 26.64 | 4.91 | |
| Laoqimai | 23.35 | 15.24 | 55.21 | 1.61 | 27.07 | 11.38 | 35.56 | 2.21 | |
| Hanzhongbai | 32.66 | 10.25 | 26.56 | 4.25 | 35.11 | 9.18 | 22.12 | 5.33 | |
| Huangguaxian | 34.16 | 11.27 | 27.92 | 3.08 | 44.90 | 9.86 | 18.58 | 3.41 | |
| Youmangsaogudan | 47.14 | 10.43 | 18.72 | 3.65 | 39.97 | 10.79 | 22.84 | 3.49 | |
| Shengen | 33.65 | 7.52 | 18.90 | 6.24 | 36.70 | 7.67 | 17.68 | 7.89 | |
| Youzimai | 25.94 | 10.42 | 33.98 | 5.50 | 35.21 | 10.91 | 26.23 | 6.20 | |
| Chushanbao | 39.84 | 11.70 | 24.84 | 3.27 | 32.92 | 9.90 | 25.45 | 3.17 | |
| Wumangchunmai | 34.85 | 10.04 | 24.39 | 2.43 | 39.11 | 10.22 | 22.11 | 2.22 | |
| Huoliaomai | 85.12 | 8.53 | 8.48 | 1.26 | 71.11 | 9.31 | 11.08 | 1.51 | |
| Jiangdongmen | 47.78 | 9.02 | 15.97 | 7.38 | 55.41 | 7.88 | 12.03 | 8.06 | |
| Daqingmang | 51.14 | 11.08 | 18.33 | 5.18 | 52.74 | 9.43 | 15.13 | 5.56 | |
| Dabaimai | 36.42 | 10.93 | 25.40 | 6.76 | 34.76 | 11.32 | 27.56 | 7.00 | |
| Naolaohan | 30.75 | 11.31 | 31.11 | 1.77 | 36.01 | 10.78 | 25.33 | 2.48 | |
| Shanmai | 38.78 | 10.31 | 22.50 | 1.28 | 40.42 | 12.20 | 25.53 | 1.63 | |
| Baiqitou | 33.44 | 10.37 | 26.24 | 2.34 | 41.09 | 9.40 | 19.35 | 2.21 | |
| Yangmai | 41.56 | 11.37 | 23.14 | 2.54 | 51.94 | 10.54 | 17.18 | 2.71 | |
| Chejianzi | 40.23 | 8.51 | 17.90 | 1.68 | 50.92 | 9.23 | 15.33 | 1.97 | |
| Rumai | 41.38 | 8.37 | 17.12 | 8.98 | 37.87 | 8.68 | 19.40 | 7.80 | |
| Sankecun | 44.59 | 8.93 | 16.95 | 4.83 | 54.78 | 8.99 | 13.88 | 4.92 | |
| Tumangmai | 30.05 | 12.05 | 33.94 | 5.37 | 38.17 | 10.12 | 22.43 | 5.46 | |
| Hongmangzi | 27.83 | 9.97 | 30.32 | 2.24 | 38.94 | 10.29 | 22.36 | 2.23 | |
| Geerhongmai | 37.98 | 11.66 | 25.98 | 2.54 | 36.43 | 10.88 | 25.27 | 2.31 | |
| Hongheshangtou | 30.74 | 11.84 | 32.60 | 2.76 | 37.63 | 9.87 | 22.19 | 2.20 | |
| Baimaizi | 34.15 | 10.49 | 26.00 | 3.86 | 34.98 | 11.12 | 26.91 | 3.12 | |
| Jiangmai | 40.50 | 11.11 | 23.21 | 3.27 | 40.44 | 9.95 | 20.82 | 3.48 | |
| Hongxumai | 39.93 | 9.79 | 20.75 | 2.36 | 68.82 | 10.98 | 13.50 | 2.60 | |
